# Production of viable trout offspring derived from frozen whole fish

**DOI:** 10.1038/srep16045

**Published:** 2015-11-02

**Authors:** Seungki Lee, Shinsuke Seki, Naoto Katayama, Goro Yoshizaki

**Affiliations:** 1 Department of Marine Biosciences, Tokyo University of Marine Science and Technology, Tokyo 108-8477, Japan; 2 Biological and Genetic Resources Assessment Division, National Institute of Biological Resources, Incheon 404-708, Korea

## Abstract

Long-term preservation of fish fertility is essential for the conservation of endangered fishes. However, cryopreservation techniques for fish oocytes and embryos have not yet been developed. In the present study, functional eggs and sperm were derived from whole rainbow trout that had been frozen in a freezer and stored without the aid of exogenous cryoprotectants. Type A spermatogonia retrieved from frozen-thawed whole trout remained viable after freezing duration up to 1,113 days. Long-term-frozen trout spermatogonia that were intraperitoneally transplanted into triploid salmon hatchlings migrated toward the recipient gonads, where they were incorporated, and proliferated rapidly. Although all triploid recipients that did not undergo transplantation were functionally sterile, 2 of 12 female recipients and 4 of 13 male recipients reached sexual maturity. Eggs and sperm obtained from the salmon recipients were capable of producing donor-derived trout offspring. This methodology is thus a convenient emergency tool for the preservation of endangered fishes.

A number of salmonid species are experiencing rapid population decline and several species are already extinct. Long-term preservation of fish fertility is, therefore, increasingly important for the conservation of endangered fish species. The most common method to preserve genetic resources is raising live individuals in captivity. However, this strategy involves several risks, including facility accidents, infectious disease outbreaks^1^,^2^, genetic drift^3^, and the reduced fitness within natural habitats of individuals raised in captivity^4^,^5^. Cryopreservation of fish eggs or embryos would be a valuable preservation tool, but suitable techniques have not yet been developed due to the large size and high lipid content of these materials^6^.

The authors of the present study previously demonstrated a surrogate broodstock technology^7^,^8^,^9^,^10^ in which immature germ cells isolated from a target fish species are transplanted into a closely related species, so that the surrogate species can produce the eggs and sperm of target species. We also established a method for producing functional eggs and sperm derived from cryopreserved trout spermatogonia using allogeneic surrogates^11^. If frozen-thawed spermatogonia were capable of differentiating into both eggs and sperm in recipients of different species, it would be possible to generate endangered fish species through interspecies transplantation of thawed spermatogonia whenever the need arose. However, previously established protocols^11^ are rather complicated and difficult to apply in emergency cases, such as when an endangered fish species maintained in captivity unexpectedly dies. Therefore, a simple and foolproof method to preserve the genetic resources of endangered fish is urgently required.

The simplest procedure we can imagine is freezing whole fish in a freezer without any manipulations. However, unlike plants and invertebrates^12^,^13^,^14^,^15^,^16^, frozen vertebrates cannot be revived using current technology, because frozen animal cells tend to lose their functional integrity during freezing and thawing through the lethal formation of intracellular ice. As an alternative, it has been shown that viable mammals can be generated from materials frozen without cryoprotection using intracytoplasmic sperm injection (ICSI)^17^,^18^,^19^,^20^ or somatic cell nuclear transfer (SCNT)^21^,^22^,^23^. Unfortunately, ICSI requires functional eggs to produce live animals and the use of SCNT results in animals that are nuclear-cytoplasmic hybrids^24^. Therefore, both of these techniques are impractical for the conservation of endangered fish species because maternally inherited cytoplasmic compartments, including mitochondrial DNA, cannot be preserved^24^. This limitation could be overcome with the use of surrogate broodstock technology^7^,^8^,^9^,^10^,^11^ if it were possible to retrieve viable cells from frozen whole fish.

In the present study, we successfully produced functional eggs and sperm derived from spermatogonia retrieved from whole rainbow trout kept in a freezer for at least 738 days through allogeneic and xenogeneic spermatogonial transplantation.

## Results

### Optimization of the whole trout freezing process

**Figure 1:**
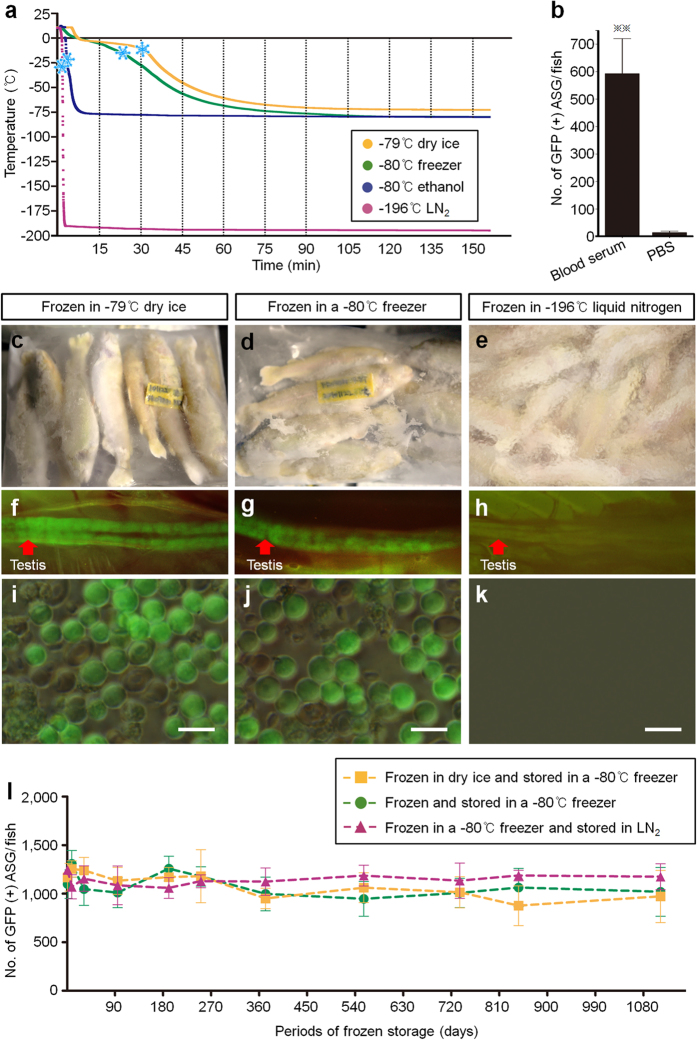
Optimization of the conditions for freezing whole trout. (**a**) Temperature changes inside whole trout during different freezing protocols: −79 °C dry ice (DI), −80 °C deep freezer, −80 °C ethanol, and −196 °C liquid nitrogen (LN_2_) (*n* = 5). Temperatures of extracellular ice formation are shown with asterisks. (**b**) Viability of type A spermatogonia (ASG) in testes slowly frozen with trout blood serum or PBS. (*n* = 5, **P < 0.01). (**c–e**) Whole trout frozen without cryoprotectants in DI (**c**), a freezer (**d**), and LN_2_ (**e**). (**f–k**) Testis and testicular cells of whole trout frozen in DI (**f**,**i**), a freezer (**g**,**j**), or LN_2_ (**h**,**k**). (**l**) Viability of ASG retrieved from whole trout frozen in DI or a freezer and then stored in a freezer or in LN_2_ for 1–1,113 days (*n* = 5–6). Data are shown as mean ± SEM. Scale bars, 20 μm (**i–k**). All images were taken by Seungki Lee.

First, we investigated the viability of type A spermatogonia (ASG) retrieved from frozen whole trout. Slow freezing, which avoids the lethal formation of intracellular ice, is widely used to maintain high cellular viability after freezing^25^. We measured the temperature changes inside the body cavities of whole trout when they were cooled under a variety of conditions (Supplementary Fig. 1). When cooled at −79 °C in dry ice (DI), at −80 °C in a freezer, at −80 °C in an ethanol bath and at −196 °C in liquid nitrogen (LN_2_), the cooling rates in the intraperitoneal cavities of whole rainbow trout were −1.0 °C/min, −1.3 °C/min, −19.8 °C/min, and −130.1 °C/min, respectively (Fig. 1a). Using these cooling protocols, extracellular ice formation (EIF) occurred at −13.8 °C, −17.2 °C, −22.5 °C, and −27.1 °C, respectively (asterisks in Fig. 1a and Supplementary Fig. 2). These results indicate that slow freezing through the EIF temperatures of −13.8 °C and −17.2 °C can be achieved by cooling whole fish in DI or a freezer.

Cryoprotectants, such as dimethyl sulfoxide, have generally been used for cryopreservation^11^,^26^. In order to establish a protocol to obtain live ASG from frozen whole fish, it is unrealistic to immerse whole fish in exogenous cryoprotectants before freezing. Therefore, in the next experiments, we investigated whether viable ASG can be obtained from testes frozen without an exogenous cryoprotectant. Since the components of extracellular fluid are nearly the same as those of blood serum, whole testes were slowly frozen in trout blood serum. PBS was used as a negative control. The viability of ASG frozen in trout blood serum (592 ± 127 ASG/fish) was significantly higher than that of ASG frozen in PBS (Fig. 1b).

We then attempted to retrieve live ASG from the frozen whole bodies of rainbow trout. Whole rainbow trout weighing 26.2 g were frozen without cryoprotectants by keeping them in DI (Fig. 1c), a deep freezer (Fig. 1d), or LN_2_ (Fig. 1e) for 7 days. ASG could be retrieved from whole trout frozen using DI (1,173 ± 182 ASG/fish) (Fig. 1i) or frozen in a deep freezer (1,361 ± 130 ASG/fish) (Fig.1j), but no viable ASG could be retrieved from whole trout frozen in LN_2_ (Fig. 1k).

To assess the continued viability of frozen ASG upon storage, fish frozen in DI or a deep freezer were stored for periods of 1, 7, 30, 94, 191, 251, 372, 556, 735, 846, and 1,113 days, and then the number of viable ASG retrieved from the frozen-thawed whole trout was determined. The viability of ASG obtained from fish frozen and stored in either DI (viability at 1 day, 1,168 ± 143 ASG/fish and at 1,113 days, 972 ± 269 ASG/fish) or a deep freezer (viability at 1 day, 1,098 ± 148 ASG/fish and at 1,113 days, 1,019 ± 251 ASG/fish) did not change significantly with increasing storage duration (Fig. 1l). The number of viable ASG obtained from whole trout stored in LN_2_ after being frozen in a freezer was not also significantly different after storage periods of 1–1,113 days (Fig. 1l). Furthermore, viable ASG could be retrieved from frozen whole trout weighing 18.8, 101.6, and 203.9 g that had remained frozen for periods of 8, 372, and 735 days (Supplementary Fig. 3f–i). However, no viable ASG was retrieved from frozen whole trout weighing 0.9 g (Supplementary Fig. 3e,i).

### Transplantation of spermatogonia retrieved from frozen whole trout

**Figure 2:**
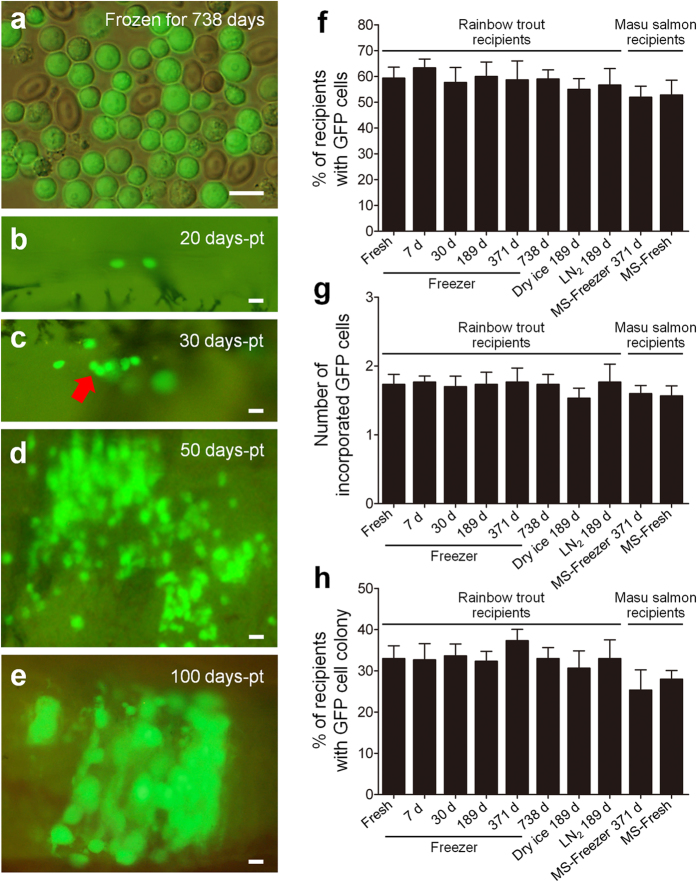
Transplantation of testicular cells retrieved from frozen whole trout. (**a**) Testicular cells retrieved from whole trout frozen in a freezer for 738 days. (**b**) Donor-derived type A spermatogonia (ASG) showing green fluorescence were incorporated into recipient trout gonads. (**c–e**) Incorporated ASG (arrow) began to proliferate (**c**), formed colonies within a recipient gonad (**d**), and started oogenesis within a female trout recipient (**e**). (**f–h**) Percentage of trout recipients that contained ASG within their gonads (**f**), number of ASG incorporated into the recipient gonads (**g**), and percentage of trout recipients with ASG colonies (**h**) were not significantly different among recipients that received ASG frozen for 7, 30, 189 (189 d, dry ice 189 d (frozen in dry ice and kept in a freezer for 189 days), and LN_2_ 189 d (frozen in a freezer and kept in LN_2_ for 189 days)), 371, and 738 days, as well as freshly prepared ASG (fresh). Similar values were obtained in salmon recipients that received ASG frozen for 371 days (MS-Freezer 371 d) and freshly prepared ASG (MS-Fresh). Data are shown as mean ± SEM (*n* = 21–45). Scale bars, 20 μm (**a–e**).

To investigate whether ASG retrieved from long-term-frozen whole trout (Fig. 2a) can be incorporated and resume proliferation in recipient gonads, the transplantation efficiencies of ASG frozen for 7, 30, 189, 371, and 738 days were compared with those of freshly prepared ASG. Recipients were dissected at 20, 30, 50, 100 (only for trout), and 153 (only for salmon) days post-transplantation (pt), and the behavior of donor-derived ASG was observed. At 20 days pt, intraperitoneally transplanted ASG had migrated toward, and were incorporated into, the genital ridges of recipients regardless of the length of freezing periods (Fig. 2b,f). The frequency of recipients carrying donor-derived ASG in their genital ridges and the mean number of ASG incorporated into recipient genital ridges did not show any significant differences among the different freezing periods and the freshly prepared control (Fig. 2f,g). ASG frozen for 738 days began to proliferate rapidly between 30 and 50 days pt (Fig. 2c,d) and formed colonies within the recipient gonads in 31 of 95 recipients (Fig. 2d,h). These values remained essentially constant among the different freezing periods and the freshly prepared control. At 100 days pt, GFP-positive oocytes, which were derived from frozen donor ASG, began oogenesis within the ovaries of trout recipients (Fig. 2e). Similar transplantation efficiencies were also observed using ASG retrieved from whole trout weighing 203.9 g that had been frozen for a period of 735 days (Supplementary Fig. 3j,k, and m-o).

Frozen trout ASG that were transplanted into salmon recipients also migrated toward the recipients’ genital ridges and were subsequently incorporated into them (Supplementary Fig. 4a and Fig. 2f). The transplanted donor ASG began to proliferate (Supplementary Fig. 4b,c, and Fig. 2h) and differentiated into oocytes in female recipients (Supplementary Fig. 4d). In masu salmon recipients, the colonization (Fig. 2f,g) and proliferation capacities of ASG frozen for 371 days (Fig. 2h) were not significantly different from those of freshly prepared control ASG. Furthermore, ASG retrieved from whole trout frozen in DI and then kept in a freezer for 189 days or from whole trout frozen in a freezer and kept in LN_2_ for 189 days also migrated toward and were incorporated into the recipients’ genital ridges when they were transplanted into trout hatchlings (Fig. 2f–h).

### Production of sperm derived from frozen whole trout

**Figure 3:**
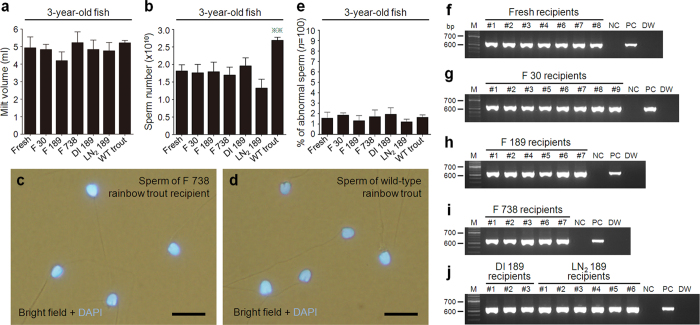
Sperm derived from frozen whole trout. (**a**,**b**) Milt volume (**a**) and sperm number (**b**) produced by recipients that received type A spermatogonia (ASG) derived from whole trout frozen for 0 (fresh), 30 (F 30), 189 (F 189, dry ice (DI) 189, and liquid nitrogen (LN_2_) 189), and 738 days (F 738), as well as wild-type trout (WT trout) at 3 years of age (**P < 0.01). (**c**) Sperm produced by a male F 738 recipient. (**d**) Sperm produced by a male WT trout. (**e**) Percentage of abnormal sperm obtained from fresh, F 30, F 189, F 738, DI 189, LN_2_ 189 recipients, and WT trout at 3 years of age. (**f–j**) PCR analyses, performed with *Gfp*-specific primers, of fresh (**f**), F30 (**g**), F 189 (**h**), F 738 (**i**), DI 189, and LN_2_ 189 milt (**j**) at 3 years of age. Lanes are labeled as follows: M, markers; numbers. 1–9, milt obtained from recipients; NC, milt obtained from WT trout; PC, milt obtained from sibling trout of donor; and DW, distilled water. Data are shown as mean ± SEM. Scale bars, 10 μm (**c**,**d**).

To confirm the production of sperm derived from frozen whole trout, experiments were conducted using male trout recipients that had received ASG retrieved from frozen orange-colored *vasa*-*Gfp* whole rainbow trout. All triploid males that had not received transplants were sterile, other than one exceptional individual that was capable of producing small amounts of aneuploid sperm (Table 1). However, 4 of 26 (15.4%), 5 of 24 (20.8%), and 5 of 23 (21.7%) males that received ASG retrieved from whole trout kept frozen in a freezer for 738 days (F 738 males) reached sexual maturity at 1, 2, and 3 years pt, respectively (Table 1). Similar tendencies were also observed in groups that received ASG retrieved from whole trout frozen for 0, 7, 30, 189, and 371 days (fresh, F 7, F 30, F 189, DI 189, LN_2_ 189, and F 371 males, respectively; Table 1). Milt volumes (0.6 ± 0.1, 2.9 ± 0.3, and 5.2 ± 0.6 ml at 1, 2, and 3 years pt, respectively) and sperm numbers (0.8 ± 0.3 × 10^9^, 0.7 ± 0.2 × 10^10^, and 1.7 ± 0.2 × 10^10^ at 1, 2, and 3 years pt, respectively) obtained from F 738 males did not significantly differ from those obtained from recipients that received ASG retrieved from whole trout frozen for shorter periods or from fresh trout of the same ages (Fig. 3a,b and Supplementary Fig. 5a–d). Furthermore, the external morphology of the sperm obtained from F 738 males (Fig. 3c) appeared similar to that of sperm obtained from wild-type (WT) trout males (Fig. 3d). The frequency of morphologically abnormal sperm was not significantly different among sperm obtained from WT trout; fresh, F 30, F 189, and F 738 males; and males that received ASG retrieved from whole trouts kept in a freezer for 189 days after freezing using DI (DI 189 males) or from whole trout stored in LN_2_ for 189 days after freezing in a freezer (LN_2_ 189 males) (Fig. 3e).

To determine whether sperm obtained from F 738 males at 1 and 2 years of age was functional, their milt was used to inseminate eggs obtained from WT trout. The fertilization rate of eggs inseminated using milt from 1-year-old F 738 males (98.1 ± 0.9%) or 2-year-old F 738 males (99.5 ± 0.5%) did not significantly differ from that of eggs inseminated with sperm derived from fresh or WT males (Supplementary Fig. 5e,g). Similarly, the hatching rate of eggs inseminated using milt from 1-year-old F 738 males (84.3 ± 4.3%) or from 2-year-old F 738 males (86.6 ± 2.8%) did not significantly differ from that of eggs inseminated using sperm derived from fresh or WT males (Supplementary Fig. 5f,h). The results obtained with F 738 males at 3 years of age are shown in the next section.

The genetic background of sperm obtained from the 3-years-old recipients was examined using PCR with *Gfp*-specific primers^27^. The results showed that all of the milt obtained from male recipients was positive for the presence of the *Gfp* gene (Fig. 3f–j). These recipients also produced *Gfp*-positive sperm when they were 1 and 2 years old. In the F1 juveniles produced by F 738 males at 1 and 2 years of age, the percentages of orange-colored fish (47.6 ± 2.0% and 46.6 ± 1.1%, respectively) and *vasa*-*Gfp* (+) fish (51.6 ± 2.8% and 49.4 ± 1.7%, respectively) were about 50% (Supplementary Table 1). In the F1 juveniles produced by recipients that received ASG retrieved from whole trout frozen for shorter periods, as well as those produced by unfrozen trout of the same ages, the donor-derived haplotypes were also transmitted to next generation following Mendelian inheritance (Supplementary Table 1).

### Production of functional eggs from frozen whole trout

**Figure 4:**
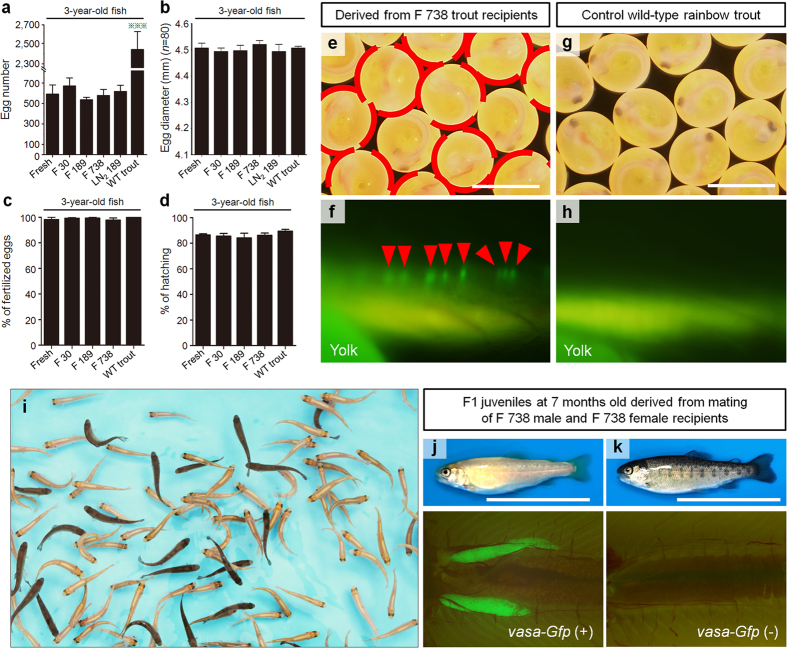
Functional eggs derived from frozen whole trout. (**a**) Numbers of eggs produced by recipients that received type A spermatogonia (ASG) retrieved from whole trout frozen for 0 (fresh), 30 (F 30), 189 (F 189 and LN_2_ 189), and 738 days (F 738), as well as wild-type trout (WT trout) at 3 years of age (***P < 0.01). (**b**) Diameter of eggs obtained from fresh, F 30, F 189, F 738, LN_2_ 189, and WT trout at 3 years of age. (**c**,**d**) Fertilization rates (**c**) and hatching rates (**d**) of eggs derived from fresh, F 30, F 189, F 738, and WT trout at 3 years of age. Eggs obtained from female recipients were inseminated with milt obtained from male recipients of each group. (**e,f**) F1 embryos derived from F 738 recipients displayed orange body color (dashed circles in **e**) and GFP-positive germ cells (arrowheads in **f**). (**g**,**h**) Embryos of WT trout as controls for **e** (**g**) and **f** (**h**). (**i–k**) Approximately 75% of F1 juveniles derived from F 738 recipients displayed the donor-derived phenotypes of orange body color (**i** and upper panel in **j**) and gonads containing GFP-positive germ cells (lower panel in **j**), suggesting that all F1 offspring were donor-derived. Phenotypes of black-pigmented body color (upper panel in **k**) and gonads containing GFP-negative germ cells (lower panel in **k**) in F1 juveniles. Data are shown as mean ± SEM. Scale bars, 5 mm (**e**,**g**); 5 cm (**j**,**k**). All images were taken by Seungki Lee.

Although none of the triploid trout females that had not received ASG matured, 5 of 25 (20.0%) and 6 of 24 (25.0%) females that received ASG retrieved from whole trout frozen for 738 days (F 738 females) reached sexual maturity at 2 and 3 years pt, respectively (Table 1). These rates were similar to those of females that received ASG derived from whole trout frozen for 0, 7, 30, 189, and 371 days (fresh, F 7, F 30, F 189, LN_2_ 189, and F 371 females, respectively; Table 1). The number of eggs ovulated by F 738 females (165 ± 26 and 579 ± 59 at 2 and 3 years pt, respectively) did not significantly differ from those obtained from recipients that received ASG derived from whole trout frozen for shorter periods and unfrozen trout of the same ages (Fig. 4a and Supplementary Fig. 6a). Furthermore, the diameters of eggs produced by F738 females were not significantly different from those produced by fresh, F 30, F 189, LN_2_ 189 recipients, or WT trout (Fig. 4b and Supplementary Fig. 6b).

To determine whether eggs ovulated by F 738 females at 2 and 3 years of age possessed normal developmental potency, the eggs produced by 2-year-old F 738 females were inseminated with milt obtained from WT trout, while the eggs from 3-year-old F 738 females were inseminated with milt obtained from F 738 males. The fertilization rates of eggs produced by 2-year-old (95.7 ± 1.3%) or 3-year-old (97.8 ± 1.6%) F 738 females were not significantly different from those obtained from eggs produced by fresh, F 30, F 189, or WT trouts (Fig. 4c and Supplementary Fig. 6c). Similarly, the hatching rates of eggs produced by 2-year-old F 738 females (84.8% ± 2.7%) or 3-year-old F 738 females (86.2% ± 1.8%) were not significantly different from those obtained from eggs produced by fresh, F 30, F 189, or WT trouts (Fig. 4d and Supplementary Fig. 6d).

F1 embryos generated from F 738 females displayed the donor-derived phenotypes of orange body color (Fig. 4e, dashed circles) and *vasa*-*Gfp*-labeled germ cells (Fig. 4f, arrowheads). In this experiment, we transplanted the ASG retrieved from dominant orange-colored (heterozygous, OR/WT) *vasa-Gfp* (hemizygous, GFP/−) frozen whole rainbow trout into WT triploid rainbow trout (WT/WT/WT, −/−/−). In the F1 juveniles produced by mating of 2-year-old F 738 females with WT trout males, the percentages of orange-colored (46.1 ± 2.6%) and *vasa*-*Gfp* (+) (48.9 ± 3.0%) fish were close to 50% (Supplementary Fig. 6e,f; Supplementary Table 2). In the F1 juveniles produced by mating of 3-year-old F 738 females and 3-year-old F 738 males, the percentages of orange-colored (75.1 ± 1.5%) and *vasa-Gfp* (+) (76.1 ± 1.6%) fish were close to 75% (Fig. 4i–k; Supplementary Table 2).

### Production of frozen whole trout-derived offspring using salmon recipients

**Figure 5:**
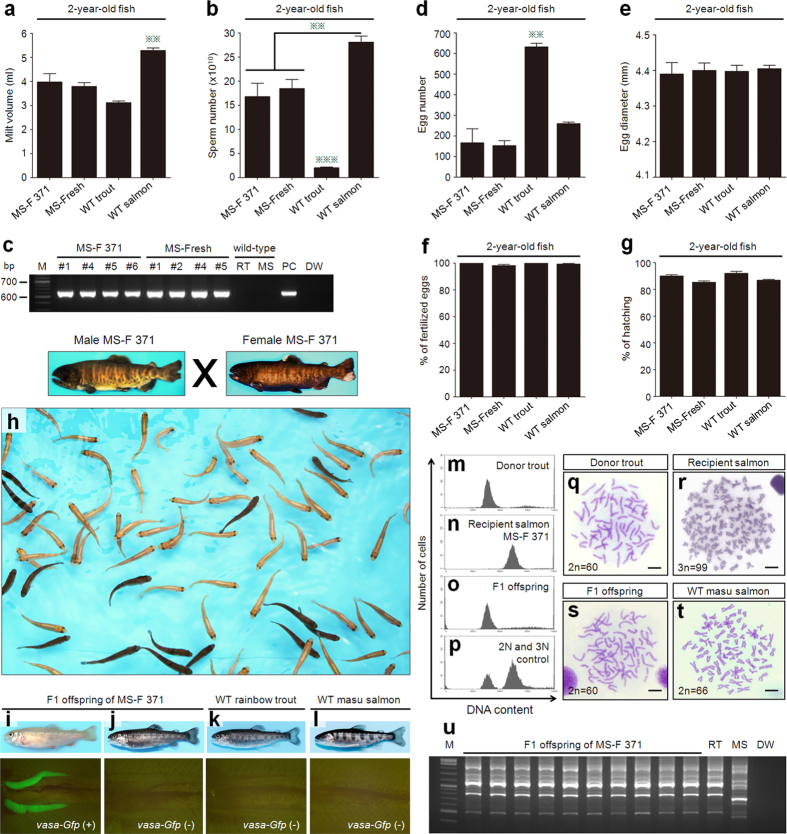
Production of frozen whole trout-derived offspring using salmon recipients. (**a**,**b**) Milt volume (**a**) and sperm number (**b**) produced by masu salmon recipients that received type A spermatogonia (ASG) retrieved from whole trout frozen for 371 days (MS-F 371), 0 days (MS-Fresh), and wild-type fish (WT trout and WT salmon) (**P < 0.01). (**c**) PCR analysis, performed with *Gfp*-specific primers, of MS-F 371, MS-Fresh, and WT milt at 2 years of age. Lanes are labeled as follows: M, marker; numbers. 1, 2, and 4–6, milt obtained from recipients; RT, milt obtained from WT rainbow trout; MS, milt obtained from WT masu salmon; PC, milt obtained from sibling trout of donor; and DW, distilled water. (**d**) Number of eggs produced by MS-F 371, MS-Fresh, WT trout, and WT salmon (**P < 0.01). (**e**) Diameter of eggs obtained from MS-F 371, MS-Fresh, WT trout, and WT salmon. (**f**,**g**) Fertilization rates (**f**) and hatching rates (**g**) of eggs derived from MS-F 371, MS-Fresh, WT trout, and WT salmon. Data are shown as mean ± SEM. **(h–l**) Approximately 75% of F1 juveniles derived from MS-F 371 salmon recipients displayed the donor-derived phenotypes of orange body color (**h**, upper panel in **i**) and gonads containing GFP-positive germ cells (lower panel in **i**). Phenotypes of black-pigmented body color (upper panel in **j**) and gonads containing GFP-negative germ cells (lower panel in **j**) in F1 juveniles derived from MS-F 371 salmon recipients. WT rainbow trout (**k**) and WT masu salmon (**l**) as controls. (**m–p**) DNA contents of a donor trout (**m**), a MS-F 371 recipient salmon (**n**), an F1 juvenile (**o**), and a mixture of diploid WT trout and triploid WT salmon (**p**). (**q–t**) Karyotypes of a donor trout (**q**), a MS-F 371 recipient salmon (**r**), an F1 juvenile (**s**), and a diploid WT salmon (**t**). (**u**) RAPD analysis of F1 juveniles produced by MS-F 371 males and females at 2 years of age. Lanes are labeled as follows: M, marker; F1 offspring of MS-F 371 recipients; RT, WT rainbow trout; MS, WT masu salmon; and DW, distilled water. All images were taken by Seungki Lee.

Two-year-old masu salmon recipients that received ASG retrieved from whole trout frozen for 371 days (MS-F 371) produced milt (4.0 ± 0.3 ml) containing numbers of sperm (16.8 ± 2.8 × 10^10^) that were equivalent to those produced by recipients that received freshly prepared ASG (Fig. 5a,b). The genetic background of the sperm obtained from these 2-year-old salmon recipients was examined using PCR with *Gfp*-specific primers^27^. The results showed that all of the milt obtained from male recipients was positive for the presence of the *Gfp* gene (Fig. 5c). Female MS-F 371 salmon recipients also produced numbers of eggs similar to those of recipients that received freshly prepared ASG and of WT salmon (Fig. 5d). Furthermore, the diameters of the eggs produced by these groups were not significantly different (Fig. 5e). Their fertilizability and hatchability were also similar (Fig. 5f,g).

In the F1 offspring produced by MS-F 371 at 2 years pt, the donor-derived haplotypes of orange body color and green fluorescence were also transmitted following Mendelian inheritance (Fig. 5h–j; Supplementary Table 2). More importantly, the external morphology of the F1 juveniles (Fig. 5i,j) was considered normal for regular rainbow trout (Fig. 5k). Analyses of the DNA content of 30 F1 juveniles produced by MS-F371 males and females revealed that all of the F1 juveniles were diploid and none of them showed any sign of aneuploidy (Fig. 5o). All of the F1 juveniles possessed the 60 chromosomes with 104 arm numbers (Fig. 5s), which is identical to the content of the donor trout (Fig. 5q) but clearly different from that of the recipient masu salmon (Fig. 5r)^28^. In addition, RAPD analysis of the F1 offspring produced by MS-F 371 showed that the DNA fingerprinting patterns of the F1 offspring were the same as those of WT rainbow trout and clearly distinct from those of the WT masu salmon (Fig. 5u).

## Discussion

The current study demonstrated that functional eggs and sperm could be derived from ASG retrieved from the testes of frozen whole fish kept in a deep freezer without the addition of cryoprotectants. Regardless of their freezing periods, ASG retrieved from frozen-thawed whole trout remained viable for at least 1,113 days. Furthermore, long-term-frozen ASG possessed the ability to differentiate into functional eggs and sperm in the ovaries and testes of allogeneic and xenogeneic triploid recipients. F1 offspring produced by salmon recipients showed the normal external morphology, *Gfp* gene expression, karyotype, and DNA fingerprint of the donor rainbow trout. Since all the triploid fish that had not received ASG could not produce any viable hatchlings, these results (donor phenotypes, cytogenetic data, and DNA fingerprint analysis) indicate that all of the gametes produced by the recipients were derived from the frozen whole trout. Thus, we could successfully produce viable offspring completely derived from ASG retrieved from frozen whole trout using both allogeneic and xenogeneic recipients.

It is well known that slow freezing and the appropriate use of cryoprotectants are key factors for the successful cryopreservation of animal cells^25^. In this study, a certain amount of body mass may work as an insulator to allow the slow freezing of ASG in the bodies of whole trout. The fact that no viable ASG could be retrieved from rapidly frozen fish in LN_2_ and small fish (0.9 g body weight) after freezing in a freezer supports this hypothesis. Nonpermeating cryoprotectants, such as sugar and proteins, are known to possess the ability to dehydrate cells and stabilize cell membranes during freezing^18^,^29^,^30^,^31^. Materials such as glucose and albumin that are present in the extracellular fluid within frozen whole trout may protect ASG from cryoinjury during freezing and thawing. Thus, the fish body working as an insulator to realize slow freezing and the trout extracellular fluid working as a cryoprotectant combine to allow ASG to remain viable after freezing whole trout in a freezer. In the present study, it was rather unexpected that the viability of ASG within frozen whole trout did not significantly decrease for at least 1,113 days in a −80 °C deep freezer. Further, long-term frozen ASG transplanted into recipients differentiated into either functional eggs or sperm offering a solution to the problem of lacking of cryopreservation techniques for fish eggs^6^.

In the present study, we could retrieve approximately 1,000 ASG from one frozen whole trout (weighing 26.2 g), and thus only 500 ASG were transplanted into each recipient. In fact, the number of ASG transplanted into each recipient was much lower than the number used in our previous experiments that used about 5,000 or 10,000 ASG per recipient^8^,^9^,^11^. Consequentially, the recipients transplanted with ASG retrieved from frozen whole trout have retained relatively low fecundity. The authors of the present study recently developed a method for improving the transplantability of ASG after short-term *in vitro* culture^32^. Combining this method with the method developed in this study would increase the fecundity of the triploid recipients.

The authors of the present study have already reported a method to produce donor-derived eggs and sperm in salmonids by transplanting ASG frozen with the aid of dimethyl sulfoxide into allogeneic recipients^11^. However, when considering the number of rapidly disappearing fish species in the world^33^, there is no time to characterize their cryobiological properties and optimize freezing protocols for each endangered species. Further, an easy and simple methodology is urgently required for emergency situations in which an endangered fish species maintained in captivity unexpectedly dies. The whole fish freezing technique developed in this study is very simple and can be widely applicable to fish weighing 18.8–203.9 g. Since the number of viable ASG retrieved in this study increased with body weight, we predicted that this protocol will also be applicable to fish heavier than 203.9 g, although further precise studies will be required.

The procedure of simply freezing whole fish can be directly applied to trout hatcheries as well as to field conditions. Indeed, since most of the trout hatcheries do not have the required laboratory apparatus, they can keep the endangered fish in a deep freezer until the endangered fish specimens can be sent to laboratories where spermatogonial transplantation is routinely performed. Therefore, this methodology of whole fish freezing is a convenient emergency tool that can be used to save endangered or even extinct fish species stored in a deep freezer.

## Methods

### Fish preparation

Dominant orange-colored (heterozygous, OR/WT)^34^ pvasa-*Gfp* transgenic (hemizygous, GFP/−)^35^,^36^ rainbow trout (*Oncorhynchus mykiss*) whose type A spermatogonia (ASG) were specifically labeled with green fluorescence^37^ were used as donors. Wild-type triploid hatchlings of rainbow trout (*O. mykiss*) (WT/WT/WT, −/−/−) at 32 days post-hatch (dph) and masu salmon (*Oncorhynchus masou*) (WT/WT/WT, −/−/−) at 37 dph were used as recipients for spermatogonial transplantation. The triploid recipients had undifferentiated primordial germ cells in the gonadal anlagen. Triploidy was induced by heat shock as previously described^9^, and the resulting triploid hatchlings were raised using 10.5 °C spring water at the Oizumi Station of Field Science Center of Tokyo University of Marine Science and Technology (Yamanashi, Japan). All experiments were approved by the Administrative Panel on Laboratory Animal Care and Use at Tokyo University of Marine Science and Technology. All methods were carried out in accordance with the Guide for the Care and Use of Laboratory Animals from Tokyo University of Marine Science and Technology.

### Freezing and thawing of whole fish

Eleven-month-old pvasa-*Gfp* transgenic rainbow trout (body weight, 24.5 ± 2.8 g; standard length, 11.8 ± 0.9 cm) anesthetized using 300 ppm 2-phenoxyethanol (Wako Pure Chemical Industries, Ltd, Tokyo, Japan) (Supplementary Fig. 1a) were cooled in a polystyrene foam box (30 × 23 × 22 cm) filled with −79 °C dry ice (DI) cubes (Supplementary Fig. 1b), in a −80 °C standard deep freezer (Supplementary Fig. 1c), in a polystyrene foam box (30 × 23 × 22 cm) filled with ethanol prechilled to −80 °C (Wako Pure Chemical Industries) using a standard deep freezer (Supplementary Fig. 1d) and in −196 °C liquid nitrogen (LN_2_) (Supplementary Fig. 1e). The temperature inside the peritoneal cavity was monitored each second for 180 min during the four different cooling protocols using a digital thermometer (Center SE-309, Center Co., Taiwan). To access the peritoneal cavity, thermocouples connected to the digital thermometer were inserted through the anus of the fish (Supplementary Fig. 1a).

To generate exothermic curves for the each cooling curve, blood samples (3 μl) prepared from 11-month-old WT rainbow trout (body weight, 22.9 ± 1.0 g; standard length, 10.5 ± 0.7 cm) were subjected to differential scanning calorimetry (Perkin Elmer Diamond DSC). Since the preceding study revealed that cooling rates in the peritoneal cavities of trout cooled in DI, a deep freezer, prechilled ethanol, and LN_2_ were −1.0 °C/min, −1.3 °C/min, −19.8 °C/min, and −130.1 °C/min, respectively, the DSC scan rates were fixed at −1.0 °C/min, −1.3 °C/min, −19.8 °C/min, and −130.1 °C/min. The detailed procedure was previously described^38^.

To examine the effects of trout blood serum as a cryoprotectant, blood serum samples from 10-month-old WT rainbow trout (body weight, 20.7 ± 1.5 g; standard length, 13.1 ± 1.1 cm) were prepared as previously described^39^. Whole testes isolated from 11-month-old pvasa-*Gfp* transgenic rainbow trout (testis weight, 0.016 ± 0.001 g) were transferred into a 2-ml cryotube (TPP, Trasadingen, Switzerland) containing 1 ml of blood serum or PBS (pH 8.2). The cryotubes were then frozen at a rate of −1 °C/min for a period of 90 min using a Bicell plastic freezing container (Nihon Freezer) located in a −80 °C deep freezer. After 24 h of frozen storage, the cryotubes were rapidly thawed in a 10 °C water bath for 2 min, and the testes were rehydrated in three changes of Eagle minimum essential medium (EMEM; Nissui) supplemented with 5% (vol/vol) FBS (Gibco), 25 mM Hepes (Sigma-Aldrich), and 2 mM l-glutamine (Sigma-Aldrich). Testes were then used to assess the viability of GFP (+) ASG.

To determine the viability of GFP (+) ASG after periods of frozen whole fish storage, 11-month-old pvasa-*Gfp* transgenic rainbow trout (body weight, 26.2 ± 3.9 g; standard length, 12.6 ± 1.0 cm) anesthetized using 300 ppm 2-phenoxyethanol (Wako) were placed into a Ziploc freezer bag (Ziploc Brand Freezer Bags, 270 × 280 mm) after water adhering to the fish was removed by using Kimtowels (Kimberly-Clark Corp.). The fish were then frozen for 3 h in either a −80 °C standard deep freezer (Fig. 1c) or a −79 °C DI cubes (Fig. 1d) and were stored at −80 °C deep freezer for given periods. For storing whole trout in −196 °C LN_2_, the frozen whole trout taken from Ziploc freezer bags after freezing in a −80 °C deep freezer or freshly anesthetized whole trout were directly plunged into −196 °C LN_2_ (Fig. 1e), before being stored in the LN_2_ tank. Whole fish frozen using these different freezing protocols were stored for 1, 7, 30, 94, 191, 251, 372, 556, 735, 846, and 1,113 days. At the appropriate times, they were completely thawed by shaking in a 10 °C water bath for at least 20 min. Testes in these frozen-thawed trout were rapidly isolated at room temperature and transferred into 10 °C EMEM supplemented with 5% (vol/vol) FBS (Gibco), 25 mM Hepes (Sigma-Aldrich), and 2 mM l-glutamine (Sigma-Aldrich) to determine the viability of GFP (+) ASG.

Further experiments were performed to examine the effect of frozen whole trout body weight on the viability of GFP (+) ASG. pvasa-*Gfp* transgenic rainbow trout weighing 0.9 ± 0.1 g at 3-month-old (Supplementary Fig. 3a), 18.8 ± 1.6 g at 10-month-old (Supplementary Fig. 3b), 101.6 ± 5.7 g at 15-month-old (Supplementary Fig. 3c), and 203.9 ± 8.0 g at 18-month-old (Supplementary Fig. 3d) were frozen in a −80 °C standard deep freezer for 8, 372, and 735 days, as described above. Frozen whole trout were then completely thawed by shaking in a 10 °C water bath for 3 min (0.9 g trout) or for at least 20 min (18.8, 101.6, and 203.9 g trout). Testes were isolated from the thawed trout as described above, and then the viability of GFP (+) ASG was examined using the method described below.

### Assessment of spermatogonial survival

Testes were minced and trypsinized for 2 h at 20 °C as previously described^8^. Dissociated testicular cells were rinsed and resuspended in EMEM supplemented with 5% (vol/vol) FBS (Gibco), 25 mM Hepes (Sigma-Aldrich), and 2 mM l-glutamine (Sigma-Aldrich). Cell suspensions were then filtered through a 42-μm pore-size nylon screen (NBC Industries) and observed under a fluorescent microscope (BX-53; Olympus). The viability of GFP (+) ASG was analyzed using the combination of a Guava PCA-96 flow cytometry system (Millipore Corporate Headquarters, Billerica, MA) and 0.4% trypan blue (Gibco BRL, Invitrogen, Rockville, MD) immediately after thawing. Because the total number of ASG per fish was almost identical among the sibling trout of the same age^11^ and cryoinjury of ASG resulted in the complete loss of *GFP* gene expression (Supplementary Table 3), the viability of the ASG is presented as the absolute number of GFP (+) and trypan blue (−) cells per fish. These values are then compared among the treatments.

### Germ cell transplantation

Testicular cell suspensions containing GFP (+) ASG were prepared from the testes (testis weight, 0.013 ± 0.001 g) of 11-month-old whole trout (body weight, 25.0 ± 2.6 g; standard length, 11.5 ± 0.8 cm) frozen for 7, 30, 189, 371, and 738 days. The testes were composed of only ASG. Control ASG was freshly prepared from unfrozen fish. Testicular cells were rinsed and resuspended in EMEM supplemented with 5% (vol/vol) FBS (Gibco), 25 mM Hepes (Sigma-Aldrich), and 2 mM l-glutamine (Sigma-Aldrich), and then filtered through a 42-μm pore-size nylon screen (NBC Inc.). Approximately 20 nl of the cell suspension, containing approximately 500 GFP (+) ASG, were intraperitoneally transplanted into WT triploid hatchlings of rainbow trout or masu salmon as previously described^8^. Recipients were dissected at 20, 30, 50, 100 (only for trout), and 153 days post-transplantation (pt) (only for salmon), and their gonads were observed under a fluorescent microscope (BX-53; Olympus). The numbers of recipients with GFP (+) ASG and numbers of GFP (+) ASG incorporated into recipient gonads were examined at 20 days pt. The numbers of recipients with GFP (+) ASG colonies were determined at 50 days pt.

Spermatogonial transplantation was further performed using GFP (+) ASG retrieved from the testes (testis weight, 0.505 ± 0.074 g) of rainbow trout weighing 203.9 ± 8.0 g at 18-month-old (Supplementary Fig. 3d) that had been frozen in a −80 °C freezer for 735 days, along with freshly prepared control ASG. The testes contained cysts of type B SG in addition to ASG. Testicular cell suspensions were prepared as described above and then transplanted into hatchlings of WT triploid rainbow trout as previously described^8^. The numbers of recipients with GFP (+) ASG and the numbers of ASG incorporated into recipient gonads were examined at 20 days pt. The numbers of recipients with proliferating GFP (+) ASG were evaluated at 30 days pt.

### Progeny Tests

Recipient fish were reared until sexual maturity. The numbers of gametes obtained from the recipients and gamete quality, including the developmental potency of the resulting F1 embryos, were determined as previously described^11^. To determine the production of sperm derived from frozen whole trout, total genomic DNA was extracted from the milt obtained from male recipients and subjected to PCR with *Gfp*-specific primers^27^. To evaluate the production of offspring by gametes derived from frozen whole trout, milt or eggs obtained from 1- and 2-year-old rainbow trout recipients were used for fertilization with eggs or milt produced by WT trout, respectively. Further, milt obtained from 1-year-old masu salmon recipients were used to inseminate WT trout eggs. Two-year-old salmon males and females were mated with each other, and three-year-old trout males and females were also mated with each other. If F1 offspring were derived from donor frozen trout (OR/WT and GFP/−), they would be expected to exhibit a 50% (recipients × WT trout) or 75% (recipients × recipients) ratio of donor phenotypes (OR and GFP) following Mendelian inheritance. To identify genotypes of rainbow trout and masu salmon, RAPD analysis was performed according to the method of Takeuchi *et al*.^7^.

### Cytogenetic analysis

To determine the ploidy level of the donor, recipients and F1 offspring, blood cells were fixed in 70% (vol/vol) ethanol and incubated for 8 h in PBS (pH 7.8) that contained RNase A (10 μg/ml; Sigma) and propidium iodide (200 μg/ml; Sigma). DNA contents were analyzed using a Guava PCA-96 flow cytometry system (Millipore). Mitotic chromosomes were made from fin and kidney cells of the donor and F1 offspring, respectively. Cells were treated with 0.4% (wt/vol) colchicine (Sigma) for 5 h, hypotonized in 0.075 M KCl (Gibco), fixed in methanol/glacial acetic acid (vol/vol; 3:1), air dried, and stained with 10% (vol/vol) Giemsa (Sigma) for 15 min as previously described^40^. For each specimen, at least 30 countable metaphase chromosomes were examined under a light microscope (BX-53; Olympus).

### Statistical analysis

All data are shown as the mean ± SEM. Statistical significance was analyzed with one-way analysis of variance (ANOVA) followed by Tukey’s multiple comparisons test. Student’s *t*-test was used for comparisons between two groups of blood serum and PBS. The statistical significance level was determined at the *P* < 0.05 level using GraphPad Prism v5.0 software (GraphPad Software Inc., San Diego, CA, USA).

## Additional Information

**How to cite this article**: Lee, S. *et al*. Production of viable trout offspring derived from frozen whole fish. *Sci. Rep*. **5**, 16045; doi: 10.1038/srep16045 (2015).

## Supplementary Material

Supplementary Information

## Figures and Tables

**Table 1:** Maturation of triploid recipients through transplantation of testicular cells taken from frozen whole trout.

Group	Freezing temperature (°C)	Storage temperature (°C)	Storage days	Donor/Recipient	No. of recipient	No. of mature/survived fish (%)					
						Male			Female		
						1 year	2 years	3 years	1 year	2 years	3 years
Fresh^a^	–	–	–	Trout/Trout	45	5/22 (22.7)	7/20 (35.0)	7/19 (36.8)	0/19 (0.0)	5/17 (29.4)	4/14 (28.6)
F 7^b^	– 80	– 80	7	Trout/Trout	48	4/19 (21.1)	5/15 (33.3)	–	0/24 (0.0)	2/23 (8.7)	–
F 30^b^	– 80	– 80	30	Trout/Trout	59	4/27 (14.8)	7/21 (33.3)	8/20 (40.0)	0/26 (0.0)	4/23 (17.4)	5/21 (23.8)
F 189^b^	– 80	– 80	189	Trout/Trout	47	2/23 (8.7)	4/20 (20.0)	6/20 (30.0)	0/20 (0.0)	3/17 (17.6)	3/16 (18.8)
F 371^b^	– 80	– 80	371	Trout/Trout	41	5/19 (26.3)	6/15 (40.0)	–	0/16 (0.0)	2/13 (15.4)	–
F 738^b^	– 80	– 80	738	Trout/Trout	60	4/26 (15.4)	5/24 (20.8)	5/23 (21.7)	0/29 (0.0)	5/25 (20.0)	6/24 (25.0)
DI 189^c^	– 79	– 80	189	Trout/Trout	33	2/15 (13.3)	3/12 (25.0)	3/12 (25.0)	0/16 (0.0)	0/15 (0.0)	0/13 (0.0)
LN_2_ 189^d^	– 80	– 196	189	Trout/Trout	43	3/18 (16.7)	4/16 (25.0)	6/14 (42.9)	0/20 (0.0)	3/19 (15.8)	4/16 (25.0)
MS-F 371^e^	– 80	– 80	371	Trout/Salmon	35	3/14 (21.4)	4/13 (30.8)	–	0/15 (0.0)	2/12 (16.7)	–
MS-Fresh^f^	–	–	–	Trout/Salmon	38	3/15 (20.0)	4/12 (33.3)	–	0/20 (0.0)	4/17 (23.5)	–
WT trout^g^	–	–	–	–	64	13/30 (43.3)	25/26 (96.2)	24/24 (100)	0/28 (0.0)	13/27 (48.1)	19/24 (79.2)
WT salmon^g^	–	–	–	–	68	15/27 (55.6)	21/21 (100)	–	0/34 (0.0)	32/32 (100)	–
Triploid trout^h^	–	–	–	–	61	0/31 (0.0)	0/27 (0.0)	1/25 (4.0)^i^	0/27 (0.0)	0/25 (0.0)	0/24 (0.0)
Triploid salmon^h^	–	–	–	–	55	0/26 (0.0)	0/23 (0.0)	0/22 (0.0)	0/24 (0.0)	0/22 (0.0)	0/21 (0.0)

^a^Rainbow trout recipients received freshly prepared testicular cells.

^b^Rainbow trout recipients received testicular cells retrieved from whole rainbow trout frozen and stored in a freezer for 7, 30, 189, 371 or 738 days.

^c^Rainbow trout recipients received testicular cells retrieved from whole rainbow trout frozen in dry ice and kept in a freezer for 189 days.

^d^Rainbow trout recipients received testicular cells retrieved from whole rainbow trout frozen in a freezer and kept in liquid nitrogen for 189 days.

^e^Masu salmon recipients received testicular cells retrieved from whole rainbow trout frozen and stored in a freezer for 371 days.

^f^Masu salmon recipients received freshly prepared testicular cells.

^g^Wild-type diploid fish that did not undergo transplantation.

^h^Wild-type triploid fish that did not undergo transplantation.

^i^A triploid male produced small amounts of aneuploid sperm.
