# Supplementary material for: Production of viable trout offspring derived from frozen whole fish
Source: Sci Rep. 2015 Nov 2;5:16045. doi: 10.1038/srep16045 (PMC4629203; doi:10.1038/srep16045)
Supplement: Supplementary Information [file srep16045-s1.pdf]

Supplementary Information

Production of viable trout offspring derived from frozen whole fish

Seungki Lee[1,2], Shinsuke Seki[1], Naoto Katayama[1] & Goro Yoshizaki[1†]

[1]Department of Marine Biosciences, Tokyo University of Marine Science and Technology, Tokyo 108-8477, Japan

[2]Biological and Genetic Resources Assessment Division, National Institute of Biological Resources, Incheon 404-708, Korea

[†]Correspondence should be addressed to Goro Yoshizaki.

Goro Yoshizaki, Professor
Department of Marine Biosciences, Tokyo University of Marine Science and Technology
4-5-7 Konan, Minato-ku, Tokyo 108-8477, Japan
Tel: +81-3-5463-0558
Fax: +81-3-5463-0558
Email: goro@kaiyodai.ac.jp

Supplementary Figure Legends

Supplementary Figure 1. Measurement of temperature changes inside whole trout during the cooling process. (**a**) To measure temperature changes in the intraperitoneal cavity during whole fish cooling, thermocouples connected to a digital thermometer were inserted through the anus of orange colored vasa-*Gfp* rainbow trout. (**b–e**) The whole trout fish were cooled in a polystyrene foam box filled with −79°C dry ice (DI) cubes (**b**), a −80°C standard deep freezer (**c**), a polystyrene foam box filled with −80°C ethanol prechilled in a standard deep freezer (**d**), and −196°C liquid nitrogen (**e**). Freezing whole fish in DI or a −80°C freezer can reproduce the slow freezing process. All images were taken by Seungki Lee.

Supplementary Figure 2. Temperature of extracellular ice formation inside of whole trout during cooling process. (**a–d**) To determine the temperatures of extracellular ice formation (EIF), trout blood samples (3 µl) were subjected to differential scanning calorimetry at scan rates of −1.0°C/min, −1.3°C/min, −19.8°C/min, and −130.1°C/min. EIF occurred at −13.8°C (**a**), −17.2°C (**b**), −22.5°C (**c**), and −27.1°C (**d**) during cooling in −79°C dry ice, in a −80°C freezer, in −80°C ethanol, and in −196°C liquid nitrogen, respectively.

Supplementary Figure 3. Freezing conditions of whole trout following body weight. (**a–d**) To examine the effects of frozen whole trout body weight on type A spermatogonia (ASG) survival, orange-colored pvasa-*Gfp* rainbow trout weighing 0.9 ± 0.1 g at 3-month-old (**a**), 18.8 ± 1.6 at 10-month-old (**b**), 101.6 ± 5.7 g at 15-month-old (**c**), and 203.9 ± 8.0 g at 18-month-old (**d**) were frozen in a −80°C standard deep freezer for 8, 372, and 735 days (n = 5). (**e**) No viable ASG were retrieved from whole trout weighing 0.9 g frozen in a freezer for 735 days. (**f–h**) Testicular cells retrieved from whole trout weighing 18.8 g (**f**), 101.6 g (**g**), and 203.9 g (**h**) frozen in a freezer for

735 days. (**i**) Viability of ASG retrieved from whole trout weighing 18.8, 101.6, and 203.9 g frozen in a freezer for 8, 372, and 735 days. There were no significant differences in ASG viability among different freezing periods. (n = 4–5). (**j**) GFP (+) ASG retrieved from 203.9 g frozen whole trout (arrow) were incorporated into the genital ridges of wild-type trout. (**k,l**) Incorporated ASG (arrows) began to proliferate within the recipient gonads (**k**) and the gonad of a nontransplanted control (**l**). (**m–o**) Percentage of recipients that contained donor ASG within recipient gonads (**m**), number of ASG incorporated into recipient gonads (**n**), and percentage of recipients having proliferating donor ASG (**o**) were not significantly different between 203.9 g frozen trout and fresh control groups (*n* = 26–33). Data are shown as mean ± SEM. Scale bars, 5 cm (**a–d**); 20 μm (**e–h, j-l**). All images were taken by Seungki Lee.

Supplementary Figure 4. Transplantation of testicular cells retrieved from frozen whole trout into salmon recipients. (**a**) Frozen-thawed trout GFP (+) ASG that were transplanted into salmon recipients migrated toward recipient genital ridges and were subsequently incorporated into them. (**b–d**) The transplanted donor ASG began to proliferate (arrow in **b** and **c**) and differentiate into oocytes in xenogeneic female recipients (**d**). Scale bars, 20 μm (**a–d**).

Supplementary Figure 5. Sperm produced by trout (1- and 2-year-old) and salmon (1-year-old) recipients and their developmental performance. (**a,b**) Milt volume (**a**) and sperm number (**b**) produced by recipients with freshly prepared type A spermatogonia (ASG; Fresh and MS-Fresh), recipients that received ASG frozen for 7 (F 7), 30 (F 30), 189 (F 189, dry ice [DI] 189, and liquid nitrogen [LN_2] 189), 371 (F 371 and MS-F 371), and 738 days (F 738), and wild-type fish (WT trout and WT salmon) at 1 year of age (**,****P* < 0.01). (**c,d**) Milt volume (**c**) and sperm number (**d**) produced by fresh, F 7, F 30, F 189, F 371, F 738, DI 189, LN_2 189, and WT

trout at 2 years of age (***P* < 0.01). (**e,f**) Fertilization rates (**e**) and hatching rates (**f**) of eggs inseminated with milt obtained from fresh, F 7, F 30, F 189, F 371, F 738, MS-F 371, MS-Fresh, WT trout, and WT salmon at 1 year of age. (**g,h**) Fertilization rates (**g**) and hatching rates (**h**) of eggs inseminated with milt obtained from fresh, F 7, F 30, F 189, F 371, F 738, and WT trout at 2 years of age. Milt obtained from trout recipients at 1 and 2 years of age were inseminated with eggs obtained from WT trout of the same ages. There were no significant differences within each developmental stage. Data are shown as mean ± SEM (*n* = number of mature fish within each group).

Supplementary Figure 6. Eggs produced by 2-year-old trout recipients and their developmental performance. (**a**) Number of eggs produced by recipients of spermatogonia derived from whole trout frozen for 0 (fresh), 7 (F 7), 30 (F 30), 189 (F 189 and liquid nitrogen [LN_2] 189), 371 (F 371), and 738 days (F 738) and wild-type (WT) trout at 2 years of age. There were no significant differences in egg number among recipient groups, excluding WT trout (***P* < 0.01). (**b**) Diameter of eggs obtained from fresh, F 7, F 30, F 189, F 371, F 738, LN_2 189, and WT trout at ages 2 years of age. (**c,d**) Fertilization rates (**c**) and hatching rates (**d**) of eggs derived from fresh, F 7, F 30, F 189, F 371, F 738, and WT trout at age 2 years of age. Eggs obtained from female recipients were inseminated with milt obtained from WT trout. (**e–h**) Approximately 50% of the embryos produced by mating between 2-year-old F 738 females and 2-year-old WT trout males displayed orange body color (dashed circles in **e**) and GFP-positive germ cells (arrowheads in **f**), suggesting that all F1 offspring were donor-derived. Embryos of WT trout as controls for **e** (**g**) and **f** (**h**). Data are shown as mean ± SEM (*n* = number of mature fish within each group). Scale bars, 5 mm (**e,g**). All images were taken by Seungki Lee.

Supplementary Figure 1 Lee et. al.

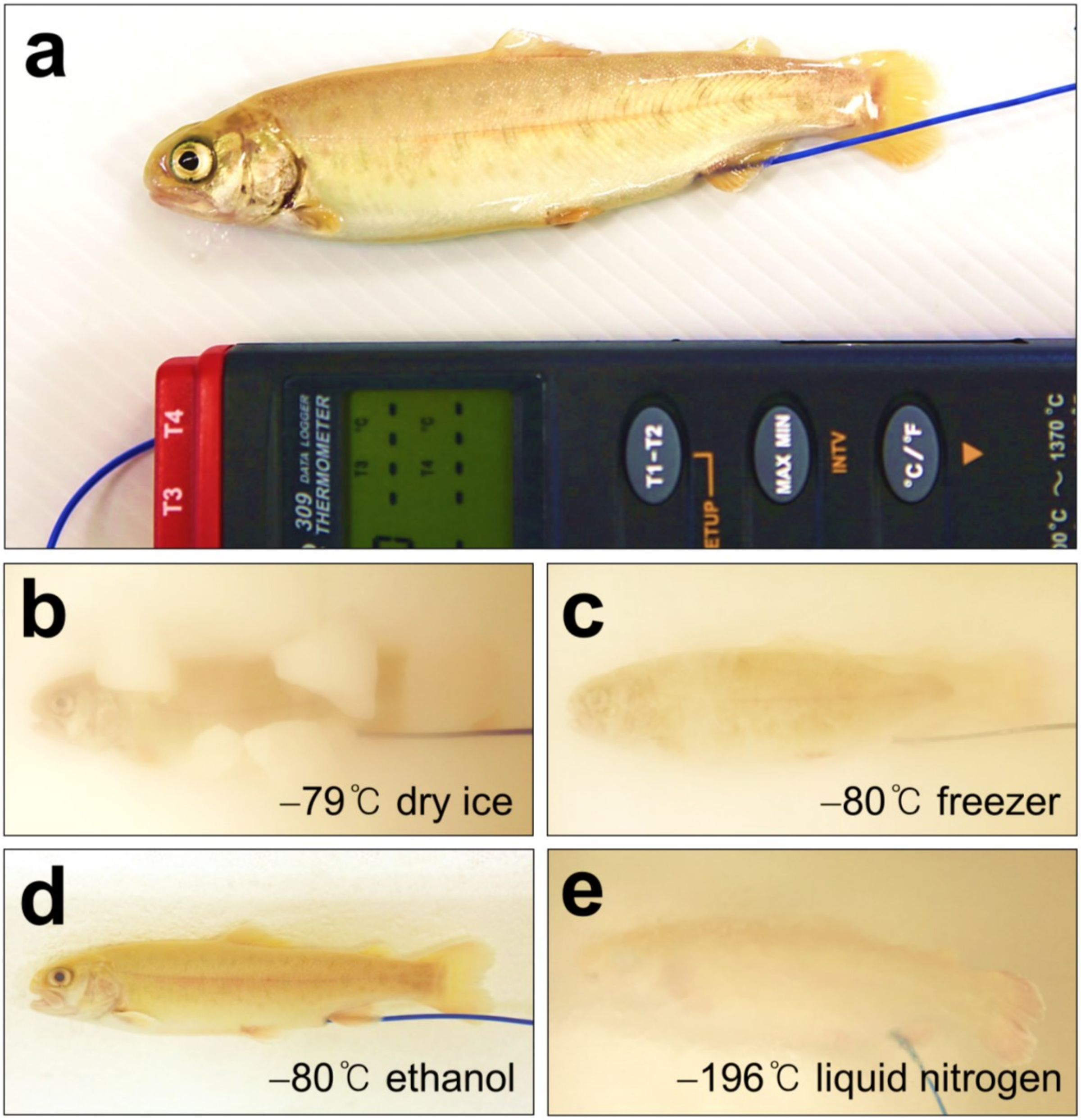

Supplementary Figure 2 Lee et. al.

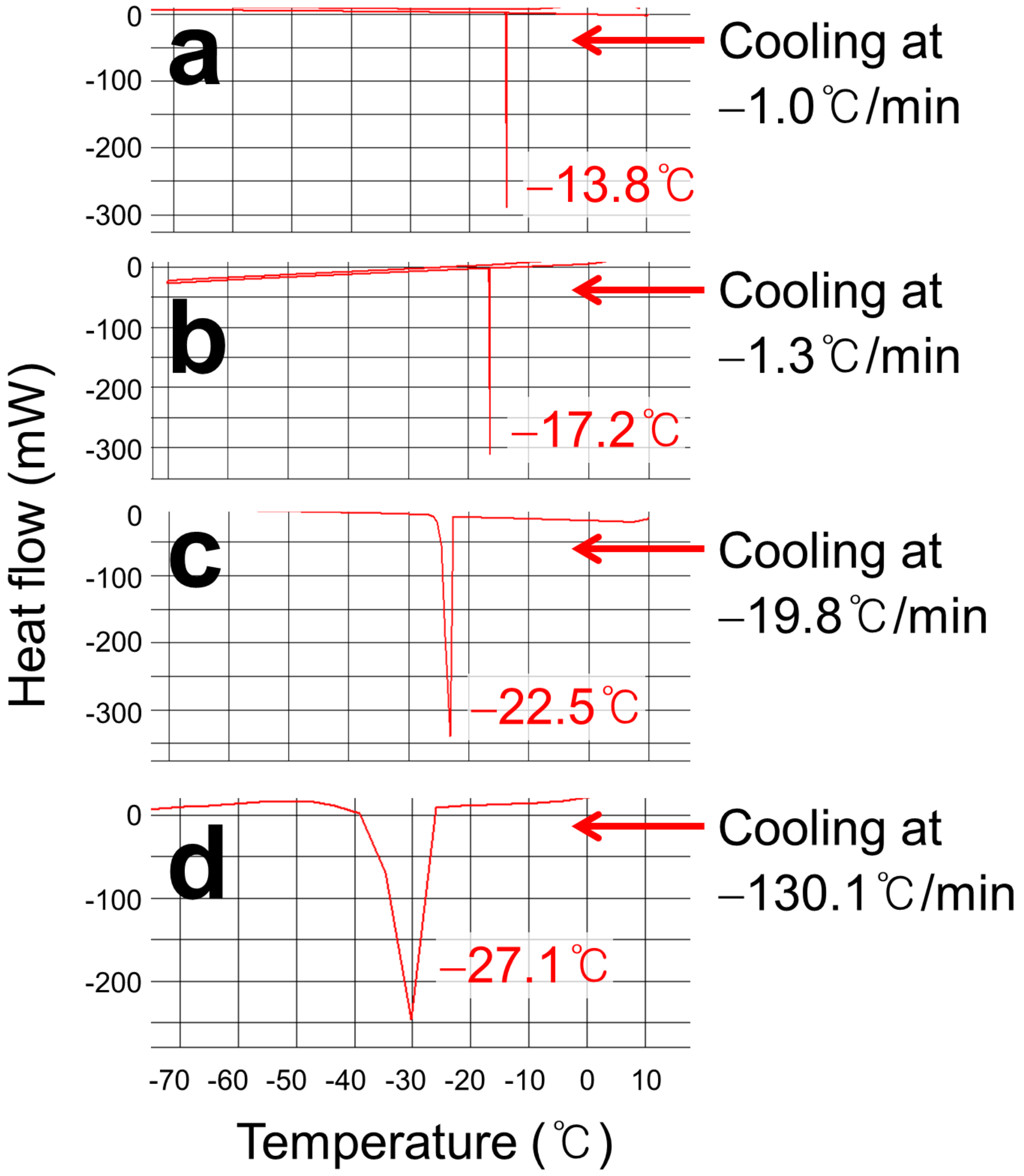

Supplementary Figure 3 Lee et. al.

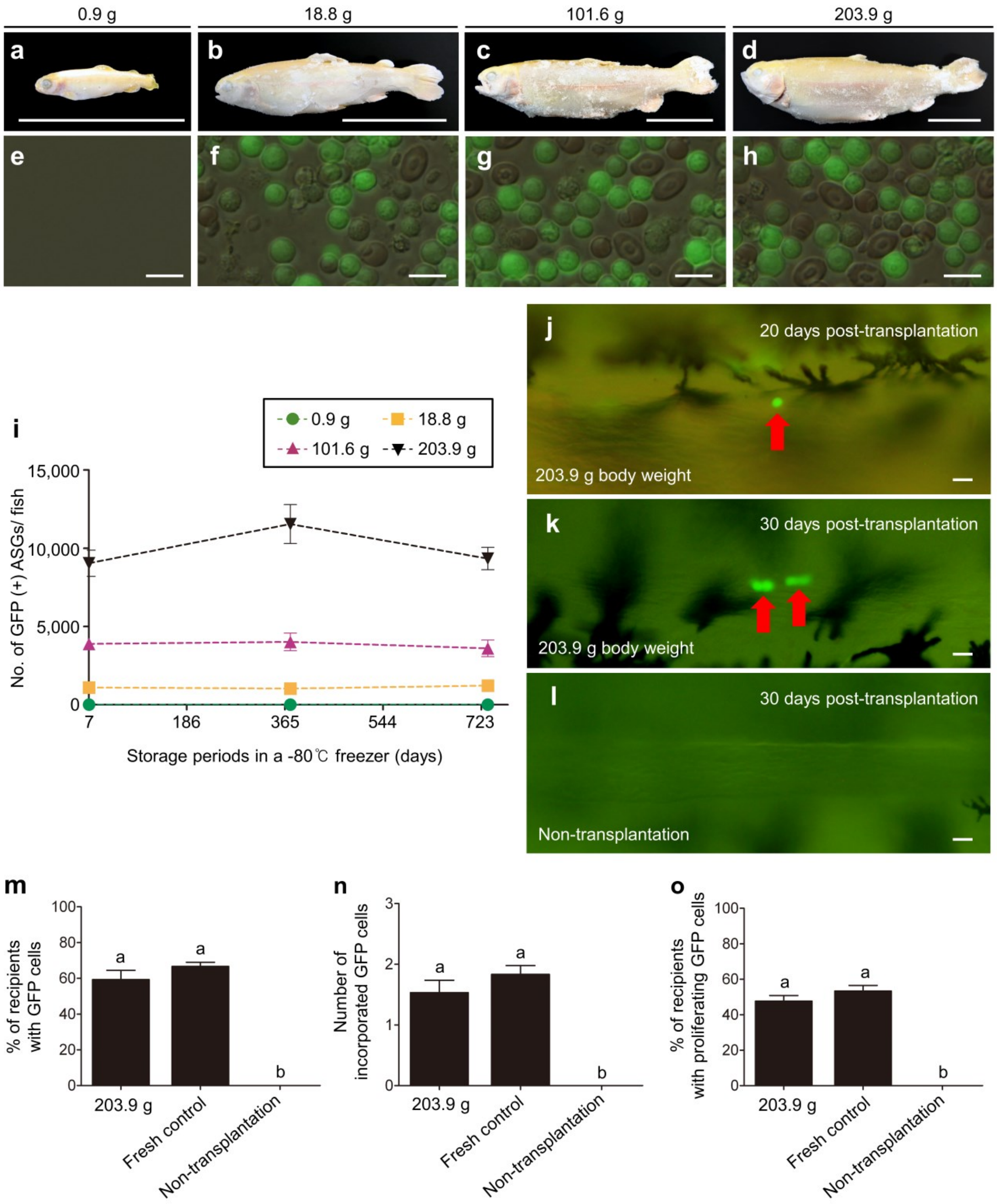

Supplementary Figure 4 Lee et. al.

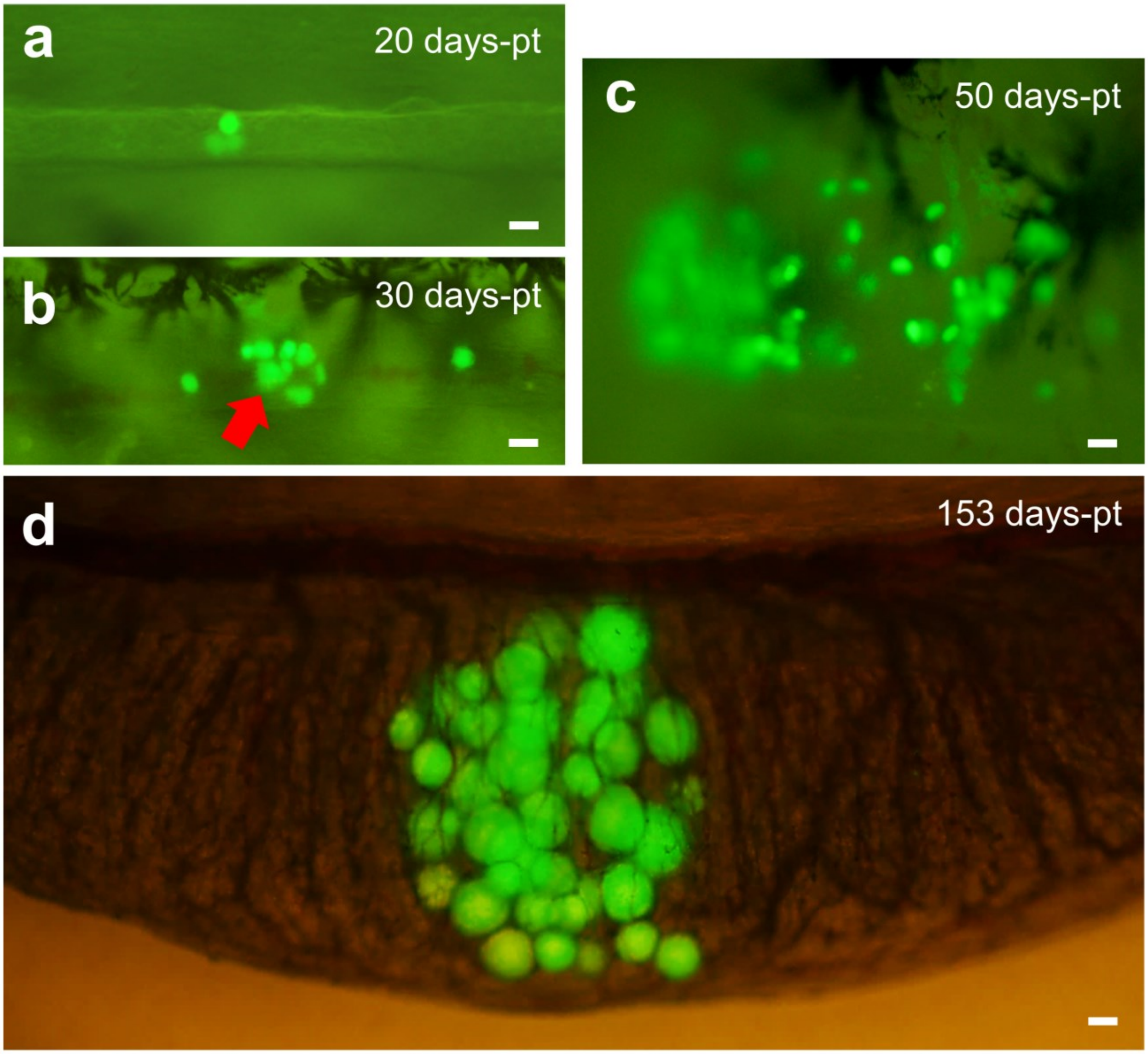

Supplementary Figure 5 Lee et. al.

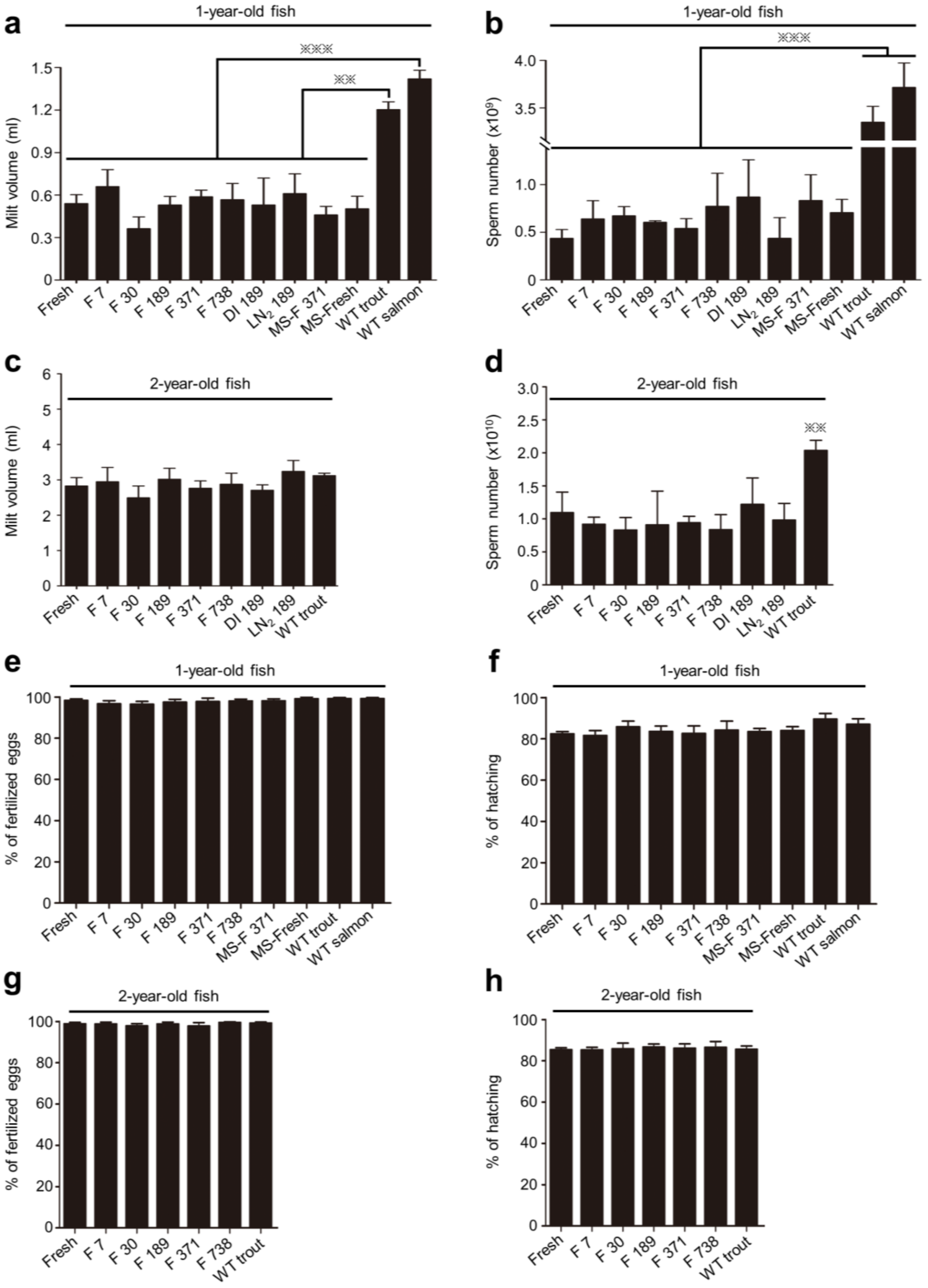

Supplementary Figure 6 Lee et. al.

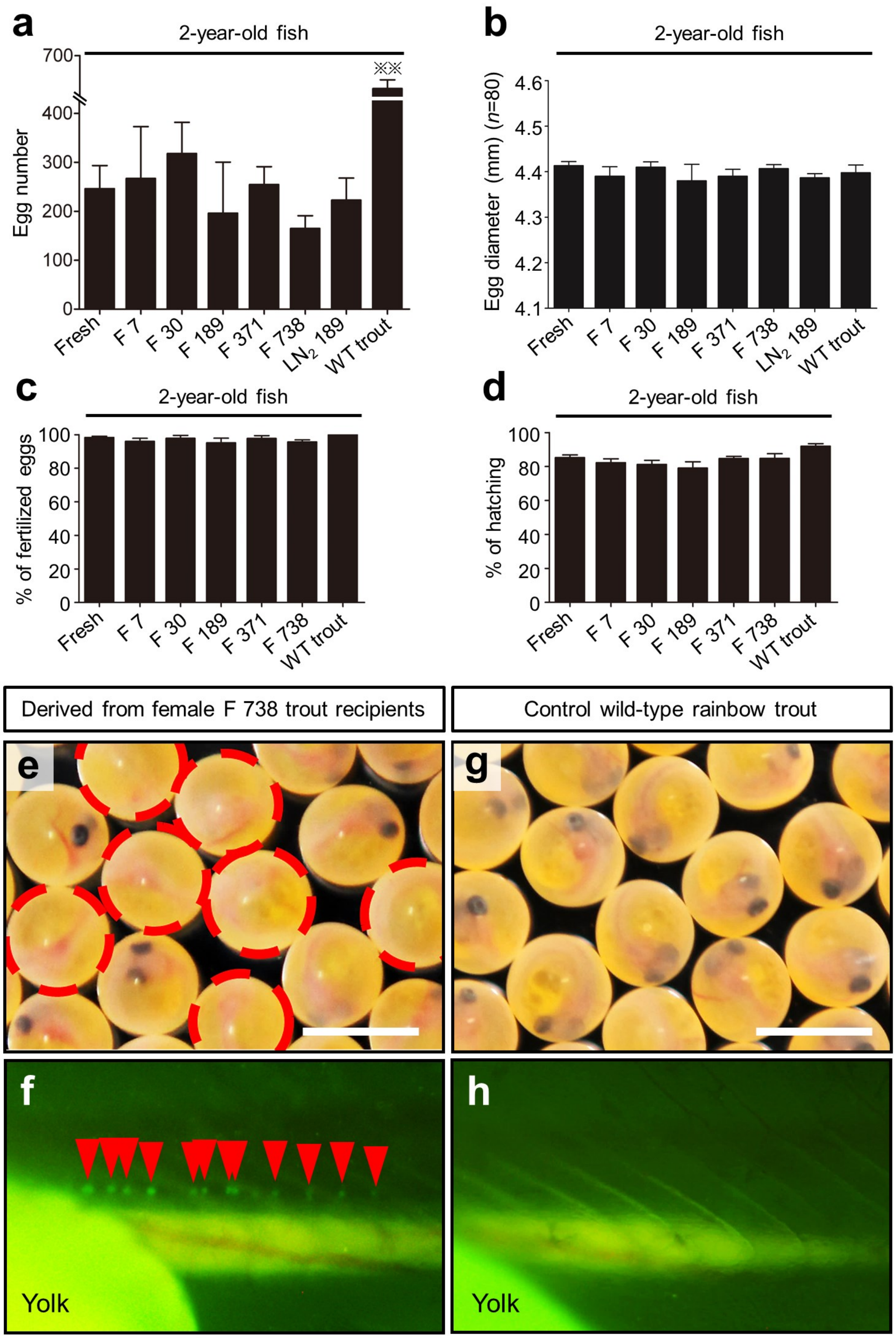

Table S1. Appearance rate of donor-derived haplotypes among F1 generation of male recipients and sex ratio in F1 offspring

Group	Male recipient	Age (year)	No. of fish analyzed	Orange-colored (%)	GFP positive (%)	Male (%)	Female (%)
Fresh[a]	#1	1	141	74 (52.5)	69 (48.9)	62 (44.0)	79 (56.0)
		2	192	91 (47.4)	102 (53.1)	97 (50.5)	95 (49.5)
	#2	1	74	34 (45.9)	43 (58.1)	38 (51.4)	36 (48.6)
		2	162	75 (46.3)	80 (49.4)	77 (47.5)	85 (52.5)
	#3	1	136	60 (44.1)	75 (55.1)	58 (42.6)	78 (57.4)
		2	208	109 (52.4)	98 (47.1)	98 (47.1)	110 (52.9)
	#4	1	90	32 (35.6)	51 (56.7)	45 (50.0)	45 (50.0)
		2	210	114 (54.3)	109 (51.9)	106 (50.5)	104 (49.5)
	#5[f]	1	93	42 (45.2)	36 (38.7)	44 (47.3)	49 (52.7)
	#6[g]	2	218	108 (49.5)	119 (54.6)	104 (47.7)	114 (52.3)
	#7[g]	2	220	99 (45.0)	105 (47.7)	100 (45.5)	120 (54.5)
	#8[g]	2	207	107 (51.7)	110 (53.1)	101 (48.8)	106 (51.2)
	Mean	1	107	48 (44.7)	55 (51.5)	49 (47.1)	57 (52.9)
		2	202	100 (49.5)	103 (51.0)	98 (48.2)	105 (51.8)
F 7[b]	#1	1	96	46 (47.9)	47 (49.0)	53 (55.2)	43 (44.8)
		2	219	108 (49.3)	118 (53.9)	104 (47.5)	115 (52.5)
	#2	1	99	49 (49.5)	59 (59.6)	47 (47.5)	52 (52.5)
		2	215	94 (43.7)	103 (47.9)	109 (50.7)	106 (49.3)
	#3	1	104	43 (41.3)	46 (44.2)	55 (52.9)	49 (47.1)
		2	193	103 (53.4)	95 (49.2)	98 (50.8)	95 (49.2)
	#4	1	87	38 (43.7)	42 (48.3)	45 (51.7)	42 (48.3)
		2	257	126 (49.0)	117 (45.5)	115 (44.7)	142 (55.3)
	#5[g]	2	252	122 (48.4)	124 (49.2)	119 (47.2)	133 (52.8)
	Mean	1	97	44 (45.6)	49 (50.3)	50 (51.8)	47 (48.2)
		2	227	111 (48.8)	111 (49.1)	109 (48.2)	118 (51.8)
F 30[b]	#1	1	116	70 (60.3)	57 (49.1)	62 (53.4)	54 (46.6)
		2	210	115 (54.8)	108 (51.4)	101 (48.1)	109 (51.9)
	#2	1	75	34 (45.3)	47 (62.7)	40 (53.3)	35 (46.7)
		2	215	104 (48.4)	109 (50.7)	113 (52.6)	102 (47.4)
	#3	1	108	58 (53.7)	50 (46.3)	55 (50.9)	53 (49.1)
		2	227	107 (47.1)	116 (51.1)	106 (46.7)	121 (53.3)
	#4	1	140	67 (47.9)	60 (42.9)	72 (51.4)	68 (48.6)
		2	253	121 (47.8)	112 (44.3)	110 (43.5)	143 (56.5)
	#5[g]	2	229	109 (47.6)	105 (45.9)	102 (44.5)	127 (55.5)
	#6[g]	2	200	105 (52.5)	97 (48.5)	107 (53.5)	93 (46.5)
	#7[g]	2	239	112 (46.9)	103 (43.1)	117 (49.0)	122 (51.0)
	Mean	1	110	57 (51.8)	54 (50.3)	57 (52.2)	53 (47.8)
		2	225	110 (49.3)	107 (47.9)	108 (48.3)	117 (51.7)

Table S1. Cont.

Group	Male recipient	Age (year)	No. of fish analyzed	Orange-colored (%)	GFP positive (%)	Male (%)	Female (%)
F 189[b]	#1	1	110	53 (48.2)	46 (41.8)	58 (52.7)	52 (47.3)
		2	214	100 (46.7)	112 (52.3)	101 (47.2)	113 (52.8)
	#2	1	133	63 (47.4)	61 (45.9)	59 (44.4)	74 (55.6)
		2	267	132 (49.4)	147 (55.1)	143 (53.6)	124 (46.4)
	#3[g]	2	198	84 (42.4)	91 (46.0)	96 (48.5)	102 (51.5)
	#4[g]	2	221	119 (53.8)	109 (49.3)	98 (44.3)	123 (55.7)
	Mean	1	122	58 (47.8)	54 (43.9)	59 (48.5)	63 (51.5)
		2	225	109 (48.1)	115 (50.7)	110 (48.4)	116 (51.6)
F 371[b]	#1	1	163	76 (46.6)	86 (52.8)	78 (47.9)	85 (52.1)
		2	228	102 (44.7)	119 (52.2)	110 (48.2)	118 (51.8)
	#2	1	216	100 (46.3)	110 (50.9)	112 (51.9)	104 (48.1)
		2	255	135 (52.9)	121 (47.5)	136 (53.3)	119 (46.7)
	#3	1	74	47 (63.5)	39 (52.7)	29 (39.2)	45 (60.8)
		2	209	104 (49.8)	113 (54.1)	102 (48.8)	107 (51.2)
	#4	1	165	80 (48.5)	76 (46.1)	83 (50.3)	82 (49.7)
		2	232	115 (49.6)	127 (54.7)	131 (56.5)	101 (43.5)
	#5[f]	1	99	43 (43.4)	49 (49.5)	53 (53.5)	46 (46.5)
	#6[g]	2	247	119 (48.2)	108 (43.7)	109 (44.1)	138 (55.9)
	#7[g]	2	200	98 (49.0)	101 (50.5)	106 (53.0)	94 (47.0)
	Mean	1	143	69 (49.7)	72 (50.4)	71 (48.6)	72 (51.4)
		2	229	112 (49.0)	115 (50.5)	116 (50.7)	113 (49.3)
F 738[b]	#1	1	82	39 (47.6)	43 (52.4)	49 (59.8)	33 (40.2)
		2	200	91 (45.5)	93 (46.5)	110 (55.0)	90 (45.0)
	#2	1	102	43 (42.2)	47 (46.1)	57 (55.9)	45 (44.1)
		2	200	90 (45.0)	99 (49.5)	89 (44.5)	111 (55.5)
	#3	1	127	65 (51.2)	62 (48.8)	61 (48.0)	66 (52.0)
		2	243	110 (45.3)	135 (55.6)	139 (57.2)	104 (42.8)
	#4[f]	1	105	52 (49.5)	62 (59.0)	54 (51.4)	51 (48.6)
	#6[g]	2	198	91 (46.0)	98 (49.5)	95 (48.0)	103 (52.0)
	#7[g]	2	210	107 (51.0)	96 (45.7)	108 (51.4)	102 (48.6)
	Mean	1	104	50 (47.6)	54 (51.6)	55 (53.8)	49 (46.2)
		2	210	98 (46.6)	104 (49.4)	108 (51.2)	102 (48.8)
MS-F 371[c]	#1	1	83	45 (54.2)	41 (49.4)	47 (56.6)	36 (43.4)
		2	237	108 (45.6)	105 (44.3)	124 (52.3)	113 (47.7)
	#2[f]	1	69	27 (39.1)	39 (56.5)	44 (63.8)	25 (36.2)
	#3[f]	1	75	33 (44.0)	34 (45.3)	30 (40.0)	45 (60.0)
	#4[g]	2	200	102 (51.0)	95 (47.5)	94 (47.0)	106 (53.0)
	#5[g]	2	285	134 (47.0)	138 (48.4)	156 (54.7)	129 (45.3)
	#6[g]	2	252	127 (50.4)	136 (54.0)	138 (54.8)	114 (45.2)
	Mean	1	76	35 (45.8)	38 (50.4)	40 (53.5)	35 (46.5)
		2	244	118 (48.5)	119 (48.5)	128 (52.2)	116 (47.8)

Table S1. Cont.

Group	Male recipient	Age (year)	No. of fish analyzed	Orange-colored (%)	GFP positive (%)	Male (%)	Female (%)
MS-Fresh[d]	#1	1	97	41 (42.3)	49 (50.5)	40 (41.2)	57 (58.8)
		2	260	122 (46.9)	120 (46.2)	117 (45.0)	143 (55.0)
	#2	1	158	78 (49.4)	83 (52.5)	80 (50.6)	78 (49.4)
		2	251	118 (47.0)	127 (50.6)	121 (48.2)	130 (51.8)
	#3[f]	1	100	45 (45.0)	52 (52.0)	55 (55.0)	45 (45.0)
	#4[g]	2	200	104 (52.0)	92 (46.0)	96 (48.0)	104 (52.0)
	#5[g]	2	200	95 (47.5)	97 (48.5)	106 (53.0)	94 (47.0)
	Mean	1	118	55 (45.5)	61 (51.7)	58 (49.0)	60 (51.0)
		2	228	110 (47.8)	109 (48.6)	110 (48.6)	118 (51.4)
WT trout[e]	Mean	1	193	0 (0.0)	0 (0.0)	91 (47.2)	102 (52.8)
	Mean	2	186	0 (0.0)	0 (0.0)	101 (54.3)	85 (45.7)
WT salmon[e]	Mean	1	183	0 (0.0)	0 (0.0)	96 (52.5)	87 (47.5)
	Mean	2	201	0 (0.0)	0 (0.0)	94 (46.8)	107 (53.2)

[a] Rainbow trout recipients received freshly prepared testicular cells.

[b] Rainbow trout recipients received testicular cells retrieved from whole rainbow trout frozen and stored in a freezer for 7, 30, 189, 371, or 738 days.

[c] Masu salmon recipients received testicular cells taken from whole rainbow trout frozen and stored in a freezer for 371 days.

[d] Masu salmon recipients received freshly prepared testicular cells.

[e] Wild-type diploid fish that did not undergo transplantation.

[f] Fish dead subsequent to spawning at age of 1 year.

[g] Fish initially matured at age of 2 years.

Table S2. Appearance rate of donor-derived haplotypes among F1 generation of female recipients and sex ratio in F1 offspring

Group	Female recipient	Age (years)	No. of fish analyzed	Orange-colored (%)	GFP positive (%)	Male (%)	Female (%)
Fresh[a]	#1	2	240	127 (52.9)	116 (48.3)	181 (75.4)	59 (24.6)
		3	352	251 (71.3)	270 (76.7)	263 (74.7)	89 (25.3)
	#2	2	200	96 (48.0)	106 (53.0)	152 (76.0)	48 (24.0)
		3	200	140 (70.0)	154 (77.0)	143 (71.5)	57 (28.5)
	#3	2	150	62 (41.3)	60 (40.0)	120 (80.0)	30 (20.0)
		3	388	289 (74.5)	272 (70.1)	285 (73.5)	103 (26.5)
	#4	2	200	94 (47.0)	98 (49.0)	138 (69.0)	62 (31.0)
		3	300	223 (74.3)	238 (79.3)	212 (70.7)	88 (29.3)
	#5[f]	2	200	92 (46.0)	95 (47.5)	130 (65.0)	70 (35.0)
	Mean	2	198	94 (47.1)	95 (47.6)	144 (73.1)	54 (26.9)
		3	310	226 (72.5)	234 (75.8)	226 (72.6)	84 (27.4)
F 7[b]	#1	2	187	90 (48.1)	99 (52.9)	147 (78.6)	40 (21.4)
	#2	2	150	71 (47.3)	78 (52.0)	124 (82.7)	26 (17.3)
	Mean	2	169	81 (47.7)	89 (52.5)	136 (80.6)	33 (19.4)
F 30[b]	#1	2	200	94 (47.0)	102 (51.0)	142 (71.0)	58 (29.0)
		3	425	317 (74.6)	331 (77.9)	340 (80.0)	85 (20.0)
	#2	2	200	104 (52.0)	95 (47.5)	157 (78.5)	43 (21.5)
		3	400	307 (76.8)	289 (72.3)	295 (73.8)	105 (26.3)
	#3	2	215	95 (44.2)	116 (54.0)	168 (78.1)	47 (21.9)
		3	338	245 (72.5)	250 (74.0)	239 (70.7)	99 (29.3)
	#4[f]	2	281	138 (49.1)	155 (55.2)	201 (71.5)	80 (28.5)
	#5[g]	3	400	299 (74.8)	312 (78.0)	281 (70.3)	119 (29.8)
	#6[g]	3	559	410 (73.3)	433 (77.5)	414 (74.1)	145 (25.9)
	Mean	2	224	108 (48.1)	117 (51.9)	167 (74.8)	57 (25.2)
		3	424	316 (74.4)	323 (75.9)	314 (73.7)	111 (26.3)
F 189[b]	#1	2	116	54 (46.6)	68 (58.6)	98 (84.5)	18 (15.5)
		3	397	308 (77.6)	280 (70.5)	287 (72.3)	110 (27.7)
	#2	2	200	102 (51.0)	95 (47.5)	145 (72.5)	55 (27.5)
		3	400	288 (72.0)	316 (79.0)	311 (77.8)	89 (22.3)
	#3	2	150	91 (60.7)	73 (48.7)	123 (82.0)	27 (18.0)
		3	400	318 (79.5)	307 (76.8)	316 (79.0)	84 (21.0)
	Mean	2	155	82 (52.7)	79 (51.6)	122 (79.7)	33 (20.3)
		3	399	305 (76.4)	301 (75.4)	305 (76.3)	94 (23.7)

Table S2. Cont.

Group	Female recipient	Age (years)	No. of fish analyzed	Orange-colored (%)	GFP positive (%)	Male (%)	Female (%)
F 371[b]	#1	2	159	72 (45.3)	89 (56.0)	112 (70.4)	47 (29.6)
	#2	2	171	89 (52.0)	85 (49.7)	122 (71.3)	49 (28.7)
	Mean	2	165	81 (48.7)	87 (52.8)	117 (70.9)	48 (29.1)
F 738[b]	#1	2	200	92 (46.0)	106 (53.0)	147 (73.5)	53 (26.5)
		3	400	292 (73.0)	318 (79.5)	322 (80.5)	78 (19.5)
	#2	2	200	94 (47.0)	108 (54.0)	155 (77.5)	45 (22.5)
		3	400	285 (71.3)	290 (72.5)	281 (70.2)	119 (29.8)
	#3	2	151	83 (55.0)	80 (53.0)	108 (71.5)	43 (28.5)
		3	576	453 (78.6)	414 (71.9)	427 (74.1)	149 (25.9)
	#4	2	60	26 (43.3)	23 (38.3)	40 (66.7)	20 (33.3)
		3	400	305 (76.3)	325 (81.3)	315 (78.8)	85 (21.2)
	#5	2	41	16 (39.0)	19 (46.3)	23 (56.1)	18 (43.9)
		3	435	347 (79.8)	320 (73.6)	339 (77.9)	96 (22.1)
	#6[g]	3	400	286 (71.5)	311 (77.8)	323 (80.2)	77 (19.3)
	Mean	2	130	62 (46.1)	67 (48.9)	95 (69.2)	36 (30.8)
		3	435	328 (75.1)	330 (76.1)	335 (77.0)	101 (23.0)
MS-F 371[c]	#1	2	181	141 (77.9)	130 (71.8)	135 (74.6)	46 (25.4)
	#2	2	82	62 (75.6)	60 (73.2)	67 (81.7)	15 (18.3)
	Mean	2	132	102 (76.8)	95 (72.5)	101 (78.1)	31 (21.9)
MS-Fresh[d]	#1	2	55	37 (67.3)	41 (74.5)	33 (60.0)	22 (40.0)
	#2	2	148	118 (79.7)	104 (70.3)	116 (78.4)	32 (21.6)
	#3	2	183	147 (80.3)	138 (75.4)	141 (77.0)	42 (23.0)
	#4	2	56	29 (51.8)	45 (80.4)	42 (75.0)	14 (25.0)
	Mean	2	111	83 (69.8)	82 (75.1)	83 (72.6)	28 (27.4)
WT trout[e]	Mean	2	241	0 (0.0)	0 (0.0)	116 (48.1)	125 (51.9)
	Mean	3	258	0 (0.0)	0 (0.0)	142 (55.0)	116 (45.0)
WT salmon[e]	Mean	2	157	0 (0.0)	0 (0.0)	85 (54.1)	72 (45.9)

[a] Rainbow trout recipients received freshly prepared testicular cells.

[b] Rainbow trout recipients received testicular cells retrieved from whole rainbow trout frozen and stored in a freezer for 7, 30, 189, 371, or 738 days.

[c] Masu salmon recipients received testicular cells taken from whole rainbow trout frozen and stored in a freezer for 371 days.

[d] Masu salmon recipients received freshly prepared testicular cells.

[e] Wild-type diploid fish that did not undergo transplantation.

[f] Fish dead subsequent to spawning at age of 2 years.

[g] Fish initially matured at age of 3 years.

Table S3. Viability of GFP-labeled type A spermatogonia on cryoinjury

Group	No. of initial GFP-labeled type A spermatogonia[c]	Freezing condition (°C)	Storage condition (°C)	Storage period (hours)	Thawing condition (°C)[d]	No. of GFP-labeled type A spermatogonia (viability, %)[e]
Frozen[a]	15,033 ± 458	Liquid nitrogen (–196)	Liquid nitrogen (–196)	3.5	Incubator (10)	0 (0)[f]
Fresh control[b]	15,467 ± 395	–	Incubator (10)	3.5	–	15,306 ± 526 (98.8 ± 1.1)[g]

[a] Type A spermatogonia with 100 ml Eagle's minimum medium (EMEM) in cryotube frozen by direct plunging into liquid nitrogen followed by slow thawing.

[b] Type A spermatogonia with 100 ml EMEM in cryotube as a control of frozen group.

[c] Initial GFP-lebeled type A spermatogonia prepared from pvasa-*Gfp* rainbow trout was maintained with EMEM in a 10°C incubator.

[d] Frozen cryotube were slowly thawed in pre-frozen Bicell slow-freezing container (Nihon Freezer) located in a 10°C incubator for 3 hours.

[e] Viability of GFP-labeled type A spermatogonia was analyzed by Guava PCA-96 flow cytometry (Millipore) immediately after thawing.

[f,g] Values in a column with different superscripts are significantly different ($P < 0.05$).

Data are shown as mean ± standard error of the mean values derived from three independent experiments.

Statistical significance was determined using Student's *t*-test for comparisons between two groups.
